# Supplementary material for: Nutrient Composition of Four Dietary Patterns in Italy: Results from an Online Survey (the INVITA Study)
Source: Foods. 2024 Jul 2;13(13):2103. doi: 10.3390/foods13132103 (PMC11240948; doi:10.3390/foods13132103)
Supplement: Supplementary file 1 [file foods-13-02103-s001.zip › foods-3071007-supplementary.pdf]

Table S1. "Missing data" for micronutrients in the food diaries (n=1410).

**Dietary Pattern**	**ME (n=348)**	**FE (n=147)**	**LOV (n=348)**	**VN (n=567)**
Iron	32	34	85	193
Calcium	1	2	12	23
Sodium	0	0	0	0
Potassium	32	40	111	216
PhosPhorus	39	47	122	263
Zinc	40	49	125	274
Magnesium	1566	767	1833	3419
Vitamin B_1	41	51	126	271
Vitamin B_2	40	48	126	271
Vitamin B_3	40	48	126	272
Vitamin B_{12}	1523	724	1728	3156
Vitamin C	12	10	30	92
Folate	41	50	129	282
Retinol eq	113	86	213	534
Beta carotene eq	83	70	187	433

Table S2. Data derived from the USDA FoodData Central (accessed 28/05/2024)

ITALIAN SEITAN IN CRUMBLES. ITALIAN SEITAN<br>Information Provided by food brand owners is label data<br>httPs://fdc.nal.usda.gov/fdc-aPP.html#/food-details/814322/nutrients<br>Data TyPe:Branded Food Category:Other Meats Brand Owner: Quisling Media Brand: Sub-Brand: FDC ID: 814322GTIN/UPC:689076762878<br>FDC Published:5/28/2020 Available Date:4/8/2020 Modified Date:4/8/2020 Market Country:United States
Veggie burgers or soyburgers. unPrePared<br>httPs://fdc.nal.usda.gov/fdc-aPP.html#/food-details/174287/nutrients<br>SR Legacy. released in APril 2018<br>Data TyPe:SR Legacy Food Category:Legumes and Legume Products FDC ID: 174287 NDB Number:16147
Vital wheat gluten<br>SR Legacy. released in APril 2018<br>httPs://fdc.nal.usda.gov/fdc-aPP.html#/food-details/168147/nutrients<br>Data TyPe:SR Legacy Food Category:Cereal Grains and Pasta FDC ID: 168147 NDB Number:48052
Soy Protein isolate<br>SR Legacy. released in APril 2018<br>httPs://fdc.nal.usda.gov/fdc-aPP.html#/food-details/174276/nutrients<br>Data TyPe:SR Legacy Food Category:Legumes and Legume Products FDC ID: 174276 NDB Number:16122
Seeds. PumPkin and squash seed kernels. roasted. without salt<br>SR Legacy. released in APril 2018<br>httPs://fdc.nal.usda.gov/fdc-aPP.html#/food-details/170557/nutrients<br>Data TyPe:SR Legacy Food Category:Nut and Seed Products FDC ID: 170557 NDB Number:12016
Seeds. sesame seeds. whole. roasted and toasted<br>SR Legacy. released in APril 2018<br>httPs://fdc.nal.usda.gov/fdc-aPP.html#/food-details/170151/nutrients<br>Data TyPe:SR Legacy Food Category:Nut and Seed Products FDC ID: 170151 NDB Number:12024

SPices. PoPPy seed<br>SR Legacy. released in APril 2018<br>httPs://fdc.nal.usda.gov/fdc-aPP.html#/food-details/171330/nutrients<br>Data TyPe:SR Legacy Food Category:SPices and Herbs FDC ID: 171330 NDB Number:2033
Barley malt flour<br>SR Legacy. released in APril 2018<br>httPs://fdc.nal.usda.gov/fdc-aPP.html#/food-details/169740/nutrients<br>Data TyPe:SR Legacy Food Category:Cereal Grains and Pasta FDC ID: 169740 NDB Number:20131
Dandelion greens. raw<br>httPs://fdc.nal.usda.gov/fdc-aPP.html#/food-details/169226/nutrients<br>SR Legacy. released in APril 2018<br>httPs://fdc.nal.usda.gov/fdc-aPP.html#/food-details/169226/nutrients<br>Data TyPe:SR Legacy Food Category:Vegetables and Vegetable Products FDC ID: 169226 NDB Number:11207
SPices. ginger. ground<br>SR Legacy. released in APril 2018<br>httPs://fdc.nal.usda.gov/fdc-aPP.html#/food-details/170926/nutrients<br>Data TyPe:SR Legacy Food Category:SPices and Herbs FDC ID: 170926 NDB Number:2021
Nuts. coconut milk. canned (liquid exPressed from grated meat and water)<br>SR Legacy. released in APril 2018<br>httPs://fdc.nal.usda.gov/fdc-aPP.html#/food-details/170173/nutrients<br>Data TyPe:SR Legacy Food Category:Nut and Seed Products FDC ID: 170173 NDB Number:12118
Beverages. rice milk. unsweetened<br>SR Legacy. released in APril 2018<br>httPs://fdc.nal.usda.gov/fdc-aPP.html#/food-details/171942/nutrients<br>Data TyPe:SR Legacy Food Category:Beverages FDC ID: 171942 NDB Number:14639

VEGAN MAYO. classic (acqua faba)
Information Provided by food brand owners is label data 2019
httPs://fdc.nal.usda.gov/fdc-aPP.html#/food-details/660064/nutrients
Data TyPe:Branded Food Category:Salad Dressing & Mayonnaise Brand Owner: Kensington & Sons LLC Brand: Sub-Brand:FDC ID: 660064 GTIN/UPC:850551005395
SUNFLOWER OIL. AQUAFABA (WATER. CHICKPEAS). DISTILLED VINEGAR. SALT. ORGANIC LEMON JUICE. ORGANIC KOMBU SEAWEED. WHITE PEPPER.

Oil. flaxseed. cold Pressed
SR Legacy. released in APril 2018
httPs://fdc.nal.usda.gov/fdc-aPP.html#/food-details/167702/nutrients
Data TyPe:SR Legacy Food Category:Fats and Oils FDC ID: 167702 NDB Number:42231

Seeds. sesame butter. tahini. from roasted and toasted kernels (most common tyPe)
SR Legacy. released in APril 2018
httPs://fdc.nal.usda.gov/fdc-aPP.html#/food-details/170189/nutrients
Data TyPe:SR Legacy Food Category:Nut and Seed Products FDC ID: 170189 NDB Number:12166

Mustard. PrePared. yellow
SR Legacy. released in APril 2018
httPs://fdc.nal.usda.gov/fdc-aPP.html#/food-details/172234/nutrients
Data TyPe:SR Legacy Food Category:SPices and Herbs FDC ID: 172234 NDB Number:2046
FDC Published:4/1/2019

SOY BUTTER
Information Provided by food brand owners is label data
httPs://fdc.nal.usda.gov/fdc-aPP.html#/food-details/481022/nutrients
FDC ID: 481022 GTIN/UPC:851653004262
FDC Published:4/1/2019 Available Date:7/14/2017 Modified Date:7/14/2017 Market Country:United States

---

Vinegar. cider
SR Legacy. released in APril 2018
httPs://fdc.nal.usda.gov/fdc-aPP.html#/food-details/173469/nutrients
Data TyPe:SR Legacy Food Category:SPices and Herbs FDC ID: 173469 NDB Number:2048

Soybean lecithin<br>SR Legacy. released in APril 2018<br>httPs://fdc.nal.usda.gov/fdc-aPP.html#/food-details/171426/nutrients<br>Data TyPe:SR Legacy Food Category:Fats and Oils FDC ID: 171426 NDB Number:4531
SILK Plain soy yogurt<br>SR Legacy. released in APril 2018<br>httPs://fdc.nal.usda.gov/fdc-aPP.html#/food-details/175227/nutrients<br>Data TyPe:SR Legacy Food Category:Legumes and Legume Products FDC ID: 175227 NDB Number:16252
Carob flour<br>SR Legacy. released in APril 2018<br>httPs://fdc.nal.usda.gov/fdc-aPP.html#/food-details/173755/nutrients<br>Data TyPe:SR Legacy Food Category:Legumes and Legume Products FDC ID: 173755 NDB Number:16055
Vermicelli. made from soy<br>SR Legacy. released in APril 2018<br>httPs://fdc.nal.usda.gov/fdc-aPP.html#/food-details/169884/nutrients<br>Data TyPe:SR Legacy Food Category:Legumes and Legume Products FDC ID: 169884 NDB Number:43114
Molasses<br>SR Legacy. released in APril 2018<br>httPs://fdc.nal.usda.gov/fdc-aPP.html#/food-details/168820/nutrients<br>Data TyPe:SR Legacy Food Category:Sweets FDC ID: 168820 NDB Number:19304

Table S3. Percent stratification of the variables.

Variables	P	ME	FE	LOV	VN
Age (yrs)	**P25**	28.00	28.00	28.00	27.00
	**P50**	32.50	35.00	33.50	36.00
	**P75**	43.25	46.00	43.00	48.00
	**P95**	60.25	60.80	59.50	64.00
Height (m)	**P25**	1.60	1.60	1.60	1.60
	**P50**	1.64	1.63	1.65	1.65
	**P75**	1.68	1.70	1.70	1.69
	**P95**	1.75	1.73	1.76	1.77
BMI (kg/m^2)	**P25**	19.60	19.15	19.68	19.57
	**P50**	21.17	20.76	20.83	21.34
	**P75**	24.01	21.99	23.22	23.31
	**P95**	28.84	26.22	29.89	28.18
Total Energy (kcal)	**P25**	1435.67	1431.33	1470.00	1434.33
	**P50**	1662.17	1634.33	1749.33	1766.00
	**P75**	1947.00	2061.00	2089.75	2057.33
	**P95**	2455.08	2531.20	2473.67	2450.93
Energy (calculated) (kcal)	**P25**	1416.91	1415.52	1472.39	1433.59
	**P50**	1658.56	1629.30	1753.51	1747.82
	**P75**	1928.32	2053.43	2061.70	2053.51
	**P95**	2467.44	2551.71	2405.43	2443.18
Total Protein (g)	**P25**	55.51	52.47	50.10	48.67
	**P50**	67.34	59.81	59.55	61.87
	**P75**	78.88	71.16	67.98	72.66
	**P95**	99.83	93.46	84.42	97.88
%Energy Protein (%)	**P25**	13.7%	13.0%	12.3%	12.2%
	**P50**	15.7%	14.4%	13.7%	14.0%
	**P75**	18.3%	16.9%	15.6%	16.7%
	**P95**	23.1%	20.7%	18.1%	21.6%

Animal Protein (g)	**P25**	8.75	2.27	0.86	0.00
	**P50**	20.13	6.97	4.77	0.00
	**P75**	34.43	13.66	9.41	0.00
	**P95**	65.27	22.85	20.42	1.27
Plant Protein (g)	**P25**	34.31	45.45	42.85	48.45
	**P50**	42.21	55.11	53.07	61.34
	**P75**	54.05	66.00	62.91	72.66
	**P95**	70.66	84.43	80.57	97.82
Total fat (g)	**P25**	41.87	41.61	43.59	41.08
	**P50**	54.35	53.95	58.49	55.60
	**P75**	66.07	74.30	76.98	69.93
	**P95**	98.73	107.87	113.21	102.20
%Energy fat (%)	**P25**	25.1%	24.3%	25.4%	23.6%
	**P50**	29.6%	28.7%	30.2%	28.8%
	**P75**	33.9%	33.5%	34.2%	34.1%
	**P95**	41.2%	42.3%	44.2%	44.8%
Animal fat (g)	**P25**	8.17	1.91	1.72	0.00
	**P50**	15.18	7.20	6.86	0.00
	**P75**	24.45	11.88	13.72	0.11
	**P95**	42.57	23.06	24.54	1.70
Plant fat (g)	**P25**	24.51	28.50	33.78	40.06
	**P50**	34.54	45.50	46.69	55.50
	**P75**	52.84	66.82	66.42	69.72
	**P95**	76.17	91.17	102.53	101.66
Total saturated fat (g)	**P25**	11.50	9.90	10.20	7.69
	**P50**	15.03	13.48	14.50	11.27
	**P75**	19.75	19.95	20.78	15.49
	**P95**	32.52	33.04	30.86	24.70
Total monounsaturated fat (g)	**P25**	16.00	16.19	16.96	14.66

	**P50**	21.87	21.32	22.94	23.16
	**P75**	29.61	29.36	34.91	32.71
	**P95**	44.06	42.69	52.82	51.34
Total Polyunsaturated fat (g)	**P25**	7.83	8.23	9.10	10.53
	**P50**	10.41	11.71	11.89	13.45
	**P75**	14.01	15.99	15.16	17.00
	**P95**	18.25	20.58	20.47	24.49
Cholesterol (mg)	**P25**	77.90	17.17	11.21	0.00
	**P50**	121.94	63.58	37.34	0.06
	**P75**	195.85	113.82	91.73	1.67
	**P95**	365.02	232.15	215.73	12.47
Total carbohydrate (g)	**P25**	184.04	203.32	202.79	197.13
	**P50**	223.36	232.07	244.06	238.07
	**P75**	262.10	263.88	278.47	289.98
	**P95**	347.78	374.19	342.01	346.20
%Energy carbohydrate (%)	**P25**	49.9%	51.1%	51.2%	51.8%
	**P50**	54.1%	55.8%	56.2%	56.3%
	**P75**	58.8%	60.0%	60.1%	60.7%
	**P95**	64.9%	68.1%	67.5%	68.6%
Starch (g)	**P25**	122.22	126.35	128.35	124.19
	**P50**	148.17	161.31	158.86	159.34
	**P75**	173.27	189.16	188.64	199.90
	**P95**	239.90	242.93	239.51	245.40
Soluble carbohydrate (g)	**P25**	55.05	53.44	55.45	51.39
	**P50**	69.75	69.51	69.06	69.47
	**P75**	86.44	87.53	88.00	89.71
	**P95**	120.15	127.65	123.64	115.93
%Energy soluble carbohydrate (%)	**P25**	13.2%	12.8%	12.4%	12.3%
	**P50**	15.6%	15.5%	15.4%	14.9%
	**P75**	18.1%	17.6%	18.5%	18.2%

	**P95**	24.0%	23.2%	23.9%	24.9%
Fiber (g)	**P25**	21.97	24.36	25.72	30.00
	**P50**	27.55	37.52	33.16	37.94
	**P75**	35.35	42.75	40.88	45.11
	**P95**	48.25	56.32	51.59	64.43
Water (g)	**P25**	1964.97	1848.53	1847.78	1839.58
	**P50**	2291.20	2425.30	2263.96	2339.52
	**P75**	2811.11	2756.28	2736.61	2848.43
	**P95**	4038.16	3291.55	3437.27	4285.21
Alcohol (g)	**P25**	0.00	0.00	0.00	0.00
	**P50**	0.01	0.05	0.02	0.04
	**P75**	3.24	1.35	2.64	0.15
	**P95**	12.16	10.08	13.78	11.90
Iron (mg)	**P25**	11.26	13.04	12.35	13.52
	**P50**	14.29	16.28	14.85	17.48
	**P75**	17.12	21.09	19.35	21.23
	**P95**	23.27	24.57	24.91	28.55
Calcium (mg)	**P25**	656.67	642.89	674.22	638.41
	**P50**	811.98	776.63	825.64	812.17
	**P75**	970.32	916.63	993.55	983.61
	**P95**	1332.55	1402.63	1395.37	1532.51
Sodium (mg)	**P25**	1199.63	958.43	1189.42	917.80
	**P50**	1694.37	1768.44	1699.66	1270.39
	**P75**	2249.28	2306.58	2326.00	1841.97
	**P95**	4018.37	3143.35	4365.77	3101.70
Potassium (mg)	**P25**	2446.50	2459.61	2401.65	2764.95
	**P50**	3059.01	3189.33	2999.32	3454.06
	**P75**	3692.73	4258.90	3695.35	4441.60
	**P95**	4977.79	5226.52	5419.57	6140.35
Phosphorus (mg)	**P25**	990.75	935.13	937.78	907.71
	**P50**	1161.50	1113.72	1085.14	1109.70

	**P75**	1339.38	1399.68	1287.31	1338.50
	**P95**	1743.06	1713.76	1604.34	1744.61
Zinc (mg)	**P25**	7.28	6.79	6.90	6.88
	**P50**	8.74	8.22	8.23	8.25
	**P75**	10.27	10.55	9.51	10.42
	**P95**	12.51	14.35	11.47	13.00
Magnesium (mg)	**P25**	281.29	263.68	263.15	273.47
	**P50**	320.20	348.43	329.74	322.39
	**P75**	368.70	414.87	403.04	439.31
	**P95**	526.21	500.87	538.94	570.35
Vitamin B_1 (mg)	**P25**	1.12	1.18	1.15	1.21
	**P50**	1.33	1.49	1.42	1.56
	**P75**	1.54	1.97	1.87	2.02
	**P95**	2.13	3.18	3.11	3.07
Vitamin B_2 (mg)	**P25**	1.14	1.05	1.04	0.98
	**P50**	1.41	1.34	1.27	1.23
	**P75**	1.73	1.65	1.50	1.47
	**P95**	2.28	2.44	2.15	1.90
Vitamin B_3 (mg)	**P25**	13.02	12.35	11.88	13.47
	**P50**	16.93	15.87	14.94	16.76
	**P75**	21.07	19.25	18.38	20.00
	**P95**	31.69	25.27	25.04	28.98
Vitamin B_{12} (mcg)	**P25**	1.07	0.33	0.25	0.00
	**P50**	2.28	1.31	0.61	0.00
	**P75**	3.33	2.13	1.31	0.60
	**P95**	6.87	7.84	2.34	1.36
Vitamin C (mg)	**P25**	85.18	84.08	77.99	97.79
	**P50**	130.83	134.03	132.51	156.74
	**P75**	181.00	189.95	189.04	222.40
	**P95**	343.38	320.57	295.14	344.42
Folate (mcg)	**P25**	293.02	340.67	325.87	386.89

	**P50**	392.69	444.73	417.59	475.70
	**P75**	499.16	624.48	497.53	586.58
	**P95**	638.84	745.55	729.30	758.96
Retinol eq (mcg)	**P25**	519.64	472.53	549.55	449.18
	**P50**	775.18	842.15	790.65	837.33
	**P75**	1304.85	1282.99	1174.49	1275.29
	**P95**	2266.00	2600.41	2240.52	2390.78
Beta carotene eq (mcg)	**P25**	2432.11	2294.96	2770.79	2698.52
	**P50**	3905.18	3924.23	4050.93	4797.34
	**P75**	7137.67	6783.66	6421.30	7654.66
	**P95**	11593.17	15608.29	12952.30	14344.74
